# Testing the attentional boundary conditions of subliminal semantic priming: the influence of semantic and phonological task sets

**DOI:** 10.3389/fnhum.2012.00241

**Published:** 2012-08-29

**Authors:** Sarah C. Adams, Markus Kiefer

**Affiliations:** Department of Psychiatry, University of UlmUlm, Germany

**Keywords:** automatic processes, masked semantic priming, unconscious visual processing, attention, task sets

## Abstract

Recent studies challenged the classical notion of automaticity and indicated that even unconscious automatic semantic processing is under attentional control to some extent. In line with our attentional sensitization model, these data suggest that a sensitization of semantic pathways by a semantic task set is necessary for subliminal semantic priming to occur while non-semantic task sets attenuate priming. In the present study, we tested whether masked semantic priming is also reduced by phonological task sets using the previously developed induction task paradigm. This would substantiate the notion that attention to semantics is necessary for eliciting unconscious semantic priming. Participants first performed semantic and phonological induction tasks that should either activate a semantic or a phonological task set. Subsequent to the induction task, a masked prime word, either associated or non-associated with the following lexical decision target word, was presented. Across two experiments, we varied the nature of the phonological induction task (word phonology vs. letter phonology) to assess whether the attentional focus on the entire word vs. single letters modulates subsequent masked semantic priming. In both experiments, subliminal semantic priming was only found subsequent to the semantic induction task, but was attenuated following either phonological induction task. These results indicate that attention to phonology attenuates subsequent semantic processing of unconsciously presented primes whether or not attention is directed to the entire word or to single letters. The present findings therefore substantiate earlier evidence that an attentional orientation toward semantics is necessary for subliminal semantic priming to be elicited.

## Introduction

The automaticity of semantic processing of written words is subject of a controversial debate: some researchers argue that semantic processing of word meaning occurs automatically in the sense that it is initiated without deliberate intention and can even occur in the absence of conscious awareness of word presentation (Greenwald et al., 1996; Kiefer and Spitzer, 2000; Kiefer, 2002, 2007; Kiefer and Martens, 2010). Others, however, propose that access to word meaning is a controlled process that depends on the intention to analyze the meaning of a word and requires conscious perception of a word (Henik et al., 1994; Duscherer and Holender, 2002). Previous evidence is contradictory and appears to support either view. As a literate adult, one would intuitively state that the understanding of the meaning of a written word happens automatically. This intuition is experimentally supported by the interference effects in the Stroop task (Stroop, 1935). In this task, participants are presented with a list of color words, which are printed in the congruent (e.g., the word “red” printed in red ink) or in the incongruent ink color (e.g., the word “red” is printed in green ink color), and are asked to name the ink color of each presented word. Naming latencies are longer in the incongruent than in the congruent condition suggesting that automatic semantic activation of the task-irrelevant word meaning interferes with the intended color naming response (McClain, 1983; Kornblum and Lee, 1995). As a consequence, response times increase the closer the irrelevant word meaning is to the color concept (Glaser and Glaser, 1989). Thus, the Stroop effect suggests that semantic processing occurs automatically as the semantic content of the words involuntarily influences responses despite the subject's intention to ignore this information and focus on color naming (Deacon and Shelley-Tremblay, 2000).

A second line of evidence favoring the automaticity of semantic processing comes from masked semantic priming studies, in which the prime is subliminally presented outside of conscious awareness. Semantic priming generally refers to the facilitation effect of a response to a target stimulus (e.g., a word) by a meaningfully related prime stimulus (Neely, 1991). Semantic priming has been frequently observed in lexical decision tasks: when subjects are asked to decide whether a target word (e.g., lemon) is a real word or a pseudoword, reaction times (RT) are faster and more accurate if the target is preceded by a semantically related word (e.g., sour) compared to an unrelated word (e.g., house). The RT difference between the semantically unrelated and related word pairs is called the semantic priming effect.

In the masked priming procedure, conscious perception of the prime is prevented by displaying a pattern mask (e.g., a random sequence of letters) after (backward masking), and frequently also before the prime (forward masking). A series of previous studies have reported consistent evidence for unconscious word processing with backward masking only (Merikle and Joordens, 1997; Daza et al., 2002; Ortells et al., 2003, 2012a). Unconscious semantic activation is demonstrated when the masked prime word facilitates processing of the target stimulus. In contrast to visible prime stimuli, for which both automatic and controlled processes usually contribute to priming effects (Neely, 1977, 1991), masked semantic priming exclusively arises from automatic preactivation of the semantic target representation by the unconsciously processed prime. Several masked semantic priming studies have shown that access to semantics can occur unconsciously in an automatic fashion (Deacon et al., 2000; Kiefer and Spitzer, 2000; Kiefer, 2002; Grossi, 2006; Kiefer and Brendel, 2006; Ortells et al., 2012b).

In contrast to this positive evidence regarding the automaticity of semantic processing, however, several researchers have argued that semantic processing does not occur automatically, but needs controlled access to conceptual meaning (Henik et al., 1994; Duscherer and Holender, 2002). This is, because semantic priming with consciously perceived stimuli strongly depends on attentional orientation toward the prime word (for reviews see, Maxfield, 1997; for reviews see, Deacon and Shelley-Tremblay, 2000). Several studies found reduced or absent semantic priming when the prime word was presented outside the focus of attention (McCarthy and Nobre, 1993; Kellenbach and Michie, 1996). Additionally, when participants were required to indicate whether a probe letter repeated above each letter of the prime word was present in the prime, semantic priming was eliminated as well (Chiappe et al., 1996). Similar results were obtained by Mari-Beffa et al. (2005), who compared a semantic categorization (living vs. non-living) and a letter search task on prime words followed by a lexical decision on the target. Compared to a semantic categorization task on the prime, which enhanced semantic priming, a letter search on the prime eliminated this robust phenomenon. Smith et al. (1983) applied the level-of processing approach to the semantic priming paradigm and examined, whether the level of prime processing influenced semantic priming. The influence of five different prime tasks (visual analysis, letter search, phonemic analysis, silent reading, and semantic analysis) on semantic priming was investigated. Significant semantic priming effects were obtained only for primes that had to be semantically and phonemically processed or just had to be silently read. In the letter search task, priming was abolished. Overall, these findings are taken as evidence that access to conceptual meaning is confined to a controlled processing mode (Henik et al., 1994; Duscherer and Holender, 2002).

These highly contradictory findings regarding the automaticity of semantic processing can be accommodated by our attentional sensitization model proposed previously (Kiefer, 2007; Kiefer and Martens, 2010). According to this model, attentional influences originating from different modulatory top down factors (e.g., attention, intention, action goals, and task sets) influence not only conscious stimulus processing, but also unconscious automatic processing. It is assumed that attentional control enhances task-relevant unconscious processes while attenuating task-irrelevant unconscious processes. Similar to attentional mechanism of conscious perception (Reynolds et al., 2000), it is supposed that control of unconscious cognition is exerted by increasing or decreasing the sensitivity of processing pathways for incoming sensory input by prefrontal top–down signals (Haynes et al., 2007). Thus, unconscious information will only be processed to the extent that it matches current attentional sets. According to our view (Kiefer and Martens, 2010), automatic semantic processing and the notion of attentional top–down control is not necessarily a contradiction as has been previously thought. This model suggests that semantic processing can occur automatically in the sense that it does not depend on conscious awareness and that it is initiated without deliberate intention. However, unlike classical models of attention and automaticity, which assume that unconscious automatic processing is independent of cognitive control, we assume that automatic semantic processing is susceptible to attentional control and requires sensitization of semantic processing pathways by a top–down signal (for a similar view, see the theoretical analysis of automaticity by Moors and De Houwer, 2006). Our notion of attentional sensitization of automatic processing therefore naturally accounts for the effects of attentional orientation on visible semantic priming described above.

In order to specifically test the proposed attentional sensitization model of unconscious cognition, top–down effects on automatic semantic processing were investigated in previous masked semantic priming experiments using a novel induction task paradigm (Martens and Kiefer, 2009; Kiefer and Martens, 2010; Martens et al., 2011). In this paradigm, participants performed two tasks in quick succession: a semantic or perceptual classification task that either induced a semantic or perceptual task set, respectively, was followed by a subliminally primed lexical decision task (word/pseudoword decision). In the semantic induction task, participants were asked to decide whether the presented word referred to a living or a non-living object. In the perceptual task, participants were requested to decide whether the letter at either the first or the last position of the presented word had a closed shape (e.g., d in doctor) or whether letters at both positions had an open shape (e.g., h and r in hammer). In line with the assumption of the attentional sensitization model, both behavioral and electrophysiological data showed reliable masked semantic priming only subsequent to the semantic induction task, but not subsequent to the perceptual task. Thus, the activated semantic task set sensitized semantic processing pathways and enhanced subliminal semantic priming whereas the perceptual task set desensitized semantic pathways and attenuated subliminal semantic priming. Similar results were obtained regardless of the difficulty level and the verbal or non-verbal nature of the induction tasks (Martens and Kiefer, 2009; Kiefer and Martens, 2010; Martens et al., 2011). Using a comparable procedure with induction trials to modulate attention, Spruyt and colleagues demonstrated that unconscious semantic priming (Spruyt et al., 2012) depends on feature-specific attention allocation. This pattern of results obtained with unconscious primes under automatic processing conditions fits nicely with previous research on attentional effects on visible prime processing. Spruyt and colleagues (Spruyt et al., 2007, 2009) also showed that feature-specific attention modulates visible semantic priming under putatively automatic processing conditions. Furthermore, as described above, prime task studies (for a review see, Maxfield, 1997) suggest that semantic priming only occurs when the consciously perceived prime is processed semantically to some extent (naming, categorizing, and silently reading), but not when the prime is processed perceptually (visual feature and letter search). Thus, visual attention to single letters or letter features clearly reduces or even abolishes semantic word processing at a conscious and unconscious level. These earlier subliminal and supraliminal priming studies therefore suggest that a semantic attentional set is necessary for semantic priming to occur. However, in these previous studies the non-semantic tasks were always perceptual and afforded attention to the shapes of single letters. Hence, it is open whether other non-semantic tasks, which require some form of linguistic processing and even attention to the entire word, similarly abolish subsequent subliminal semantic priming as the perceptual letter classification tasks.

The present study further examined the attentional boundary conditions for unconscious semantic priming to occur. We assessed whether phonological task sets that are non-semantic in their nature, but do require some form of linguistic processing also reduce unconscious semantic priming. In particular, we were interested whether the effects of the phonological induction tasks on subsequent unconscious semantic priming are comparable whether the focus is set on phonological processing of the entire word vs. single letters.

The present study therefore allows to substantiate previous findings suggesting that an attentional sensitization of semantic processing pathways by a semantic task set is necessary for eliciting unconscious semantic priming. To address these questions, we used the induction task paradigm developed previously (Kiefer and Martens, 2010; Martens et al., 2011) and contrasted in two experiments the influences of two different phonological induction tasks (phonological word vs. letter categorization) on subsequent masked semantic priming with those of a semantic induction task. The semantic or phonological induction tasks are assumed to activate a semantic or phonological task set, respectively. The induction tasks were followed in quick succession by a subliminally primed lexical decision task. Across experiments, we varied the nature of the phonological induction task in order to determine whether an attentional focus that is set either to the phonology of the entire word (Experiment 1) or to single letters/phonemes (Experiment 2) similarly influences subsequent unconscious priming. If both experiments yielded comparable results, this would demonstrate that attention to semantic vs. non-semantic stimulus dimensions (here: meaning vs. phonology), but not attention to lexical (entire word) vs. sublexical (single letters) features is the most relevant attentional factor in modulating subsequent unconscious semantic priming.

## Experiment 1

In the first experiment, we used a phonological word induction task, in which participants had to indicate whether the word begins or ends with a vowel or with a consonant. Previous research has shown that a consonant vs. vowel classification of letters of a word reliably activates phonological representations (Acha and Perea, 2010). In fact, several previous studies have used vowel/consonant classification of words to probe phonological processing (van Turennout et al., 1997, 1999; Abdel Rahman and Sommer, 2003; Abdel Rahman et al., 2003). This phonological word induction task used in our first experiment required processing of the entire word to access phonology, but did not include an attentional focus on word meaning. We expected, that even this non-semantic induction task that required lexical processing would diminish following unconscious semantic priming.

### Materials and methods

#### Subjects

Twenty-six healthy, right-handed (according to handedness test by Oldfield, 1971), native German speakers with normal or corrected-to-normal vision participated in this experiment. The data of four subjects had to be excluded due to technical problems in data acquisition. In total 22 participants (10 women and 12 men) contributed data to the experiment. They were in the age range of 20–27, with a mean age of 23.8 years. In this and the subsequent experiment, participants gave informed, written consent after the experimental task and the experimental procedure had been explained. Subjects were not aware of the purpose of the study. Both experiments were conducted in accordance with the Declaration of Helsinki.

#### Material and procedure

***Induction task.*** For the phonological word and semantic induction tasks, a stimulus set of 160 German words for each induction task were selected from an initial set of 440 German words. This selection was done on the basis of a pilot study with 12 participants (three men and nine women; average age of 24.75 years). None of these participants took part in one of the main experiments. The two induction task conditions (semantic and phonological word decision) were presented in blocks. In the phonological word induction task, half of the German words started or ended with a vowel (e.g., Apfel, Engl. apple; Hose, Engl. pants). The other half began and ended with a consonant (e.g., Kuchen, Engl. cake; Frieden, Engl. peace). The participants' task was to indicate whether the first or the last letter of the word was a vowel or whether the first and last letters were consonants. In order to correctly solve this task, participants had to read the entire word and to check the last phoneme whether it was a vowel whenever the word started with a consonant (75% of the trials). As the majority of trials required reading the entire word, this task set is most likely also applied to the remaining trials (Spruyt et al., 2007). For the semantic induction task, a different set of German words was used as stimuli. Thus, half of the words referred to living objects like e.g., pilot, dog, and carrot. The other half referred to non-living objects like e.g., bar, hammer, and cable. Stimuli for the semantic task were drawn from our earlier study (Kiefer and Martens, 2010). Here, participants were instructed to decide whether the presented word referred to a living or a non-living object. In both induction tasks, participants were told to press two assigned response buttons on a response pad with the index or middle finger as fast and as accurately as possible. Word length of all words ranged from 5 to 6 letters. Words of the different tasks were equated for word length and frequency. Task order was counterbalanced across participants. Different word sets were used for the induction tasks and the lexical decision task, in order to avoid word repetition effects.

For the final stimulus set of 80 German words in each induction task, stimulus sets were matched for response time as closely as possible in order to keep them on an equal difficulty level. Although, response times of correct answers did not significantly differ between the phonological word and the semantic induction task (743.8 vs. 696.12 ms, *p* > 0.06), the phonological word task was slightly more difficult than the semantic task. Error rates (ERs) did not show a significant difference between the semantic and phonological word induction task (2.5 vs. 3.3%, *p* > 0.07).

***Masked semantic priming paradigm.*** Stimulus material for primes and targets were identical to the ones used in earlier studies (Kiefer and Spitzer, 2000; Kiefer, 2002; Kiefer and Brendel, 2006; Martens and Kiefer, 2009; Kiefer and Martens, 2010). The set consisted of 320 German word–word and 320 word–pseudoword pairs. Primes and targets were on average five letters long (range 3–9) and subtended at a viewing distance of 90 cm a visual angle of about 2.5° in width and 0.8° in height. The word–pseudoword pairs served as distractors and were not further analyzed. The word–word pairs were composed of 160 semantically related (table–chair) and 160 semantically unrelated pairs (car-leaf). Critical prime target combinations were equated in word length and frequency (Ruoff, 1990) of the primes and of the targets across conditions (pseudowords were only matched in length). Prime and target combinations were divided in two lists. The prime target lists were combined with the induction tasks. The 80 words in each induction task were four times repeated. The allocation of a list to an experimental condition (phonological word vs. semantic induction task) was counterbalanced across subjects. In order to avoid semantic interference or priming effects from the word in the induction task to the subsequently presented prime or lexical decision target, it was assured that the inducing word was not related to the prime or the target within one trial. As the theoretical focus lied on the influence of previously induced task sets (induction task) on subsequent masked semantic priming, we were interested in possible interactions between semantic relatedness and induction task and not in the effect of semantic relatedness itself. For that reason, potential effects arising from unnoticed insufficient matching of primes and targets of the semantic relatedness conditions in linguistic variables other than word length and word frequency cannot compromise the interpretation of this theoretically relevant higher-order interaction.

**Figure 1:**
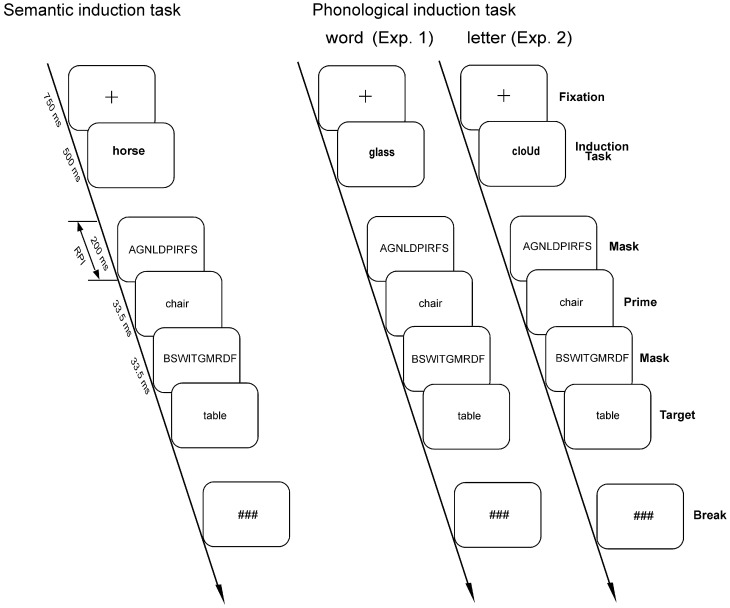
**Temporal sequence of one trial in the semantic and phonological induction task conditions of Experiments 1 and 2.** The semantic induction task in Experiments 1 and 2 required semantic classification (living/non-living object), whereas the phonological word induction task in Experiment 1 required a phonological word classification (does the word begins or ends with a vowel or begins and ends with a consonant?) and the phonological letter induction task in Experiment 2 requested a phonological letter classification (Is the uppercase letter a vowel or a consonant?). The “plus” sign indicates the fixation cross; the hash marks indicate the breaks between the trials.

The total number of 640 trials was divided into 8 blocks of 80 trials each. The stimuli of the induction task and the masked priming paradigm were combined in such a way that all conditions of the induction task and the masked priming paradigm co-occurred equally often and were entirely balanced. In order to prevent systematic response congruency effects between the induction and the lexical decision tasks, experimental conditions and response requirements (response finger) were also entirely balanced. Four subsequent blocks were allocated to each induction task (phonological word vs. semantic) in order to reduce possible influences of task switching effects. The order of the induction task blocks was counterbalanced across participants. Breaks were provided between the blocks. Figure 1 displays the sequence of events in the experimental paradigm.

In each trial, participants were first presented with a fixation cross for 750 ms, which was followed by a word for 500 ms that represented the stimulus for the induction tasks. Participants had to decide as fast and as accurately as possible (a) in the phonological word induction task, whether the word began or ended with a vowel or started and ended with a consonant, and (b) in the semantic induction task, whether the word named a living or non-living object. As soon as the response to the induction task was given, a random letter string (forward mask) consisting of 10 capital letters was presented for 200 ms (response prime interval, RPI). The random letter string was followed by the prime word, which was shown for 33.5 ms. After prime presentation, another random letter string was presented for 33.5 ms, which served as a backward mask. Thereafter, the target stimulus that either formed a real word or a pronounceable pseudoword was displayed. Participants had to decide as fast and as accurately as possible whether or not the target was a real word. Responses were indicated by pressing one of two buttons with the right index and middle finger. Participants were not informed of the presence of the prime. The target remained on the screen until a response was given. Thereafter, three hash marks were presented, which prompted the participant to initiate the next trial by pressing a button. Before the main experiment, participants were instructed and completed a practice phase with the induction task assigned to this block and the lexical decision task separately. Subsequently, they practiced the tasks in the same sequence as in the main experiment with no data sampled.

To investigate the participants' awareness of the prime between the two masks, subjects were informed of the presence of the prime word after the main experiment. All participants denied to have recognized the prime words consciously. As in our earlier priming studies (Kiefer, 2002; Kiefer and Brendel, 2006; Kiefer and Martens, 2010), an objective measure of prime identification by using a simple visual discrimination task was followed in order to have an objective assurance of the invisibility of the primes. Here, the phonological word induction task resp. the semantic induction task was combined with masked stimuli consisting of 160 words and 160 letter strings. The letter strings were composed of nine repetitions of an identical capital letter (e.g., AAAAAAAAA). Random selection of the letter strings took place in each trial. The 160 masked word stimuli were either semantically related or not to the following context word, in order to assess possible backward priming effects. The context words resembled the target words of the main experiment and were also included to keep the stimulation comparable, but were not associated with a response. As in the main experiment, the participants had first to respond to the words in the induction tasks (phonological word resp. semantic decision). Afterwards, the task was to decide whether the masked stimulus was a word or a letter string. Subjects were encouraged to make the best guess if they were unconfident about the answer. Here, accuracy was emphasized over response speed.

All stimuli were displayed in white font against a black background on a computer monitor (refresh rate = 16.67 ms). Participants were upright seated in a sound-attenuating chamber. The entire session, including instructions, the practice experiment, the main experiment and the recognition test took about 1.5 h.

### Results and discussion

#### Masked word identification test

Identification performance was distributed around the chance level of 50% (mean phonological = 52.56%, mean semantic = 53.13%), which is to be expected when merely guessing. In order to assess whether the context word facilitated identification of related masked primes (backward priming), d′ sensitivity measures for semantically related and for unrelated conditions were calculated from each participant's hit rates (correct responses to words) and false alarm rates (erroneous responses to letter strings) according to Green and Swets (1966). Backward priming would have produced a higher d′ for the related than for the unrelated condition. A repeated-measures analysis of variance (ANOVA) on d′ measures with the within-subject factors semantic relatedness and induction task revealed no significant differences between conditions, all *F*s < 2.78, *p*s > 0.11. Consequently, it can be excluded that backward priming rendered the masked prime words partially recognizable. Furthermore and most importantly, as the main effect of induction task was not significant (see above) masked prime identification was comparable for the phonological word (*d*′ = −0.06) and semantic (*d*′ = 0.23) induction tasks. As d′ in the semantic induction task condition was apparently high and significantly deviated from zero (semantic *p* < 0.02; phonological *p* > 0.66), we assessed in the semantic induction task condition whether prime visibility was related to the magnitude of priming by correlating d′ and magnitude of semantic priming (RT unrelated minus related) (Kiefer, 2002): correlation between d′ and semantic priming was close to zero (*r* = 0.03) and not significant (*p* > 0.89). We also applied a median split to divide the participants according to their prime visibility index d′ into a “low visibility” and a “high visibility” visibility group in the semantic induction task condition to further test whether partial prime visibility influenced the results (Ortells et al., 2012b). In the semantic induction task condition, the high visibility group showed a d′ mean of 0.54, which was reliably above chance, *t*_(10)_ = 4.4, *p* < 0.01, whereas the low visibility group had a d′ mean of −0.07, which did not reliably differ from zero, *t*_(10)_ = −1.67, *p* > 0.12. Additionally, a *t*-test for independent samples on semantic priming performed for group (high vs. low visibility) revealed no significant difference in the magnitude of semantic priming between groups, *t*_(20)_ = 0.82, *p* > 0.41, two-tailed. Thus, potential residual prime visibility was not associated with larger semantic priming effects.

#### Induction tasks

For RT analysis, mean RT of the correct responses was calculated for each induction task condition. Responses faster or slower than two standard deviations of the individual's means were defined as outliers and not entered into data analysis. In total, 657 trials of all participants (i.e., 4.7% of the entire data set) were excluded from analysis. Separate repeated-measures ANOVAs on mean RT and ER with the within-subject factor induction task were performed. Responses in the semantic induction task were significantly faster than responses in the phonological induction task (760.97 vs. 844.71 ms), [*F*_(1, 21)_ = 26.95, *p* < 0.001, η^2^ = 0.56]. An identical analysis of ER yielded a reversed pattern: significantly more errors were committed in the semantic induction task compared to the phonological induction task (2.1% vs. 1.4%), [*F*_(1, 21)_ = 6.21, *p* = 0.02, η^2^ = 0.23].

This RT and ER pattern in the induction tasks resembles a speed-accuracy trade-off. A speed-accuracy trade-off is, however, unlikely for two reasons. Firstly, in the pilot study ER and RTs for the phonological word induction task were non-significantly higher compared to the semantic induction task suggesting a slightly greater difficulty of the phonological word induction task. The higher ER in the semantic induction task in the main experiment can be explained by the fact that semantic judgments of word meaning are more fuzzy and error prone than phonological judgments of letters, which are based on the well-defined decision criterion whether a letter is a vowel or a consonant. This may induce errors, in particular in a demanding dual task context (for a similar result pattern see already Kiefer and Martens, 2010).

#### Masked semantic priming

**Figure 2:**
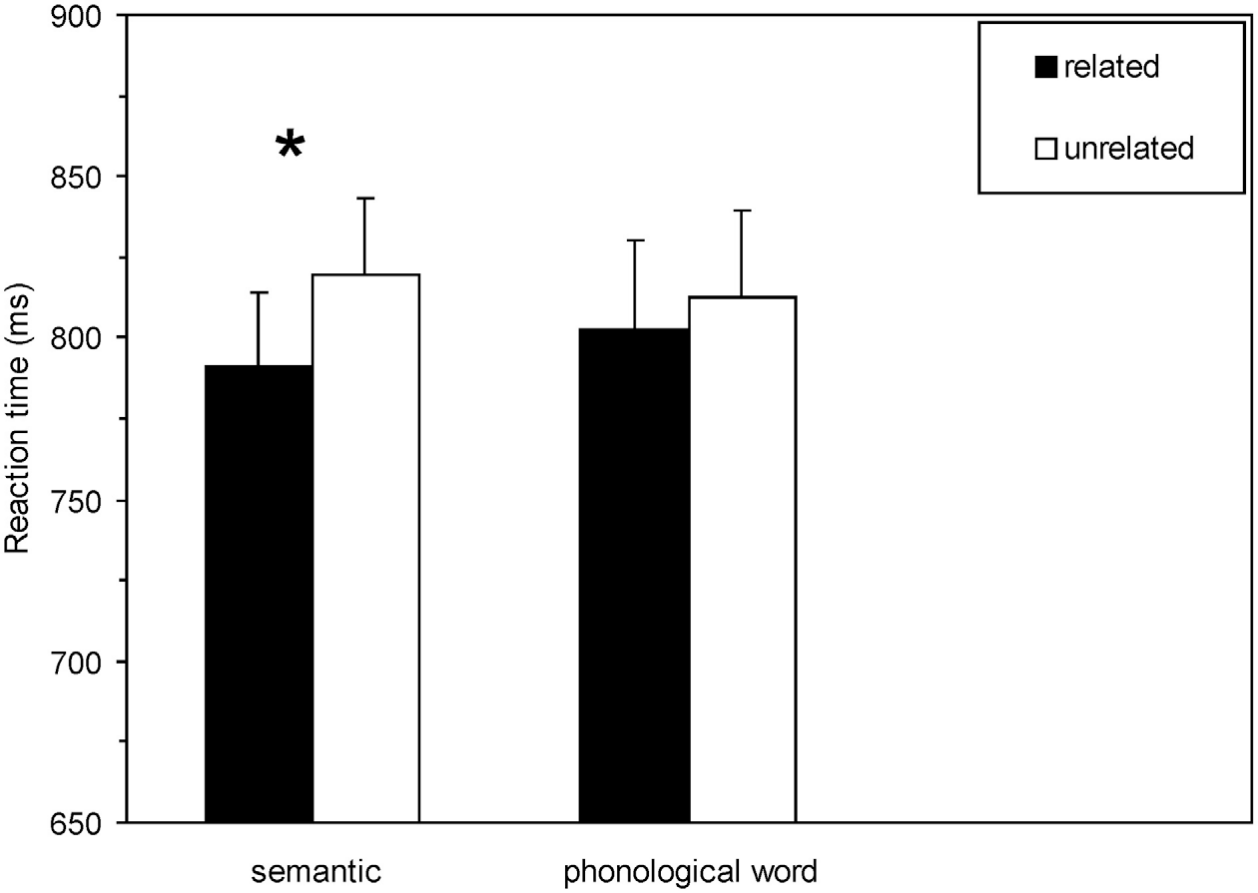
**Experiment 1.** Mean lexical decision latencies (*n* = 22) as a function of semantic relatedness and induction task. In this and the following figures, the vertical lines depict the standard error of the means of each condition, and the asterisk indicates significant masked semantic priming effects within each induction task. Related = semantically related prime target pairs; unrelated = semantically unrelated prime target pairs. The asterisk indicates significant priming effects (*p* < 0.05).

Analysis of RT data in the masked semantic priming paradigm was based on mean RT of the correct responses to target words in each experimental condition. Criteria for outlier rejection were the same as for the induction task data. 343 trials of all participants (i.e., 2.4% of the entire data set) were discarded. Separate repeated-measures ANOVAs on mean RT and ER with the within-subject factors induction task and semantic relatedness were performed. For the RT data, the main effect semantic relatedness was significant, [*F*_(1, 21)_ = 10.13, *p* < 0.0001, η^2^ = 0.33], indicating that reactions to semantically related prime target pairs were faster than to unrelated pairs (masked semantic priming effect). The main effect induction task was not significant, *p* > 0.87, demonstrating that induction tasks did not generally influence RTs in the lexical decision task. Although the interaction between the factors induction task and semantic relatedness did not reach significance, [*F*_(1, 21)_ = 1.25, *p* = 0.28], the semantic priming effect was numerically much larger subsequent to the semantic induction task (28.2 ms) compared to the phonological induction task (10.2 ms) (see Figure 2). Separate one-sample *t*-tests on semantic priming (RT difference unrelated minus related) revealed significant semantic priming only subsequent to the semantic induction task, *t*_(21)_ = 2.32, *p* = 0.03, but not following the phonological induction task, *t*_(21)_ = 1.38, *p* = 0.18. An ANOVA with the within-subject factor induction task and semantic relatedness performed on ER showed a significant effect of semantic relatedness, [*F*_(1, 21)_ = 9.47, *p* = 0.006, η^2^ = 0.31]: Participant's ERs in the lexical task were higher in the unrelated condition compared to the related condition (8.3 vs. 5.06%). There was neither a main effect of induction task nor an interaction effect between induction task and semantic relatedness—7.05 (phonological induction task) vs. 6.31% (semantic induction task), *F*s < 0.48, *p*s > 0.21.

Experiment 1 assessed whether a phonological word induction task that allocates attention to the entire word would reduce unconscious semantic priming similar to the previously investigated perceptual induction tasks that required attention to visual features of single letters (Kiefer and Martens, 2010). As predicted, masked semantic priming effects were significant only subsequent to the semantic induction task, but not subsequent to the phonological word induction task, although the interaction between semantic relatedness and induction task did not reach significance. Overall, the modulation of unconscious semantic priming by the semantic vs. non-semantic induction tasks was quite comparable to our previous study, which contrasted the influence of a semantic and perceptual letter decision induction task (Kiefer and Martens, 2010): in this earlier work, in which the interaction term was significant, we obtained 38 ms priming subsequent to the semantic induction task vs. 17 ms semantic priming subsequent to the perceptual induction task. Hence, we could basically replicate the reduction of unconscious semantic priming with a different non-semantic induction task, which required attention to the entire word. Nevertheless, this modulation of priming by previously performed semantic vs. non-semantic induction tasks was less reliable than in the previous study.

We assume that the non-significant interaction in this present experiment was due to the greater inter-subject variability of the phonological word induction task compared with the perceptual letter induction task used in the previous study (Kiefer and Martens, 2010). Probably, the phonological word induction tasks, which required attention to the entire word, encouraged participants to read the word, which in turn induced a sensitization of semantic processing in some instances: according to dual route models of reading (Riddoch et al., 1988; Seidenberg and McClelland, 1989), word reading includes both a semantic and a non-semantic route: dependent on which route is passed (semantic or non-semantic route), word reading may implicitly access semantics and consequently sensitizes semantic processing pathways, or, alternatively, word reading may obviate semantics resulting in desensitization of semantic processing pathways. Thus, if a phonological induction task requires attention to the entire word, in order to retrieve its phonological word form, sensitization and desensitization of semantic pathways may be heterogeneous across participants thereby increasing variability.

In order to reduce the variability to perform the phonological induction task, we conducted a second experiment with the same semantic induction task (living/non-living), but designed a new phonological induction task.

## Experiment 2

In the second experiment, we designed a phonological letter induction task, in which participants had to focus attention to the phonology of single letters. The subsequent subliminally primed lexical decision task was identical to Experiment 1. In the phonological letter induction task, participants were presented with words written in lower case letters with one upper case letter at different positions [e.g., kaFfee = Kaffee (Engl. coffee); hErbst = Herbst (Engl. autumn); wurzEl = Wurzel (Engl. root)]. Participants' task was to indicate, whether the upper case letter was a vowel or a consonant. As German writing is typical for its use of upper and lower case letter at the beginning of nouns, the words (all nouns) in the phonological letter induction task were highly unusually written with a lower case letter as initial letter and one upper case per word at random positions, first and final position excluded. This manipulation should allocate participants' attention to single letters and should prevent them from lexical processing and word reading (Craik and Lockhart, 1972). According to the attentional sensitization model, the task set associated with the phonological letter induction task should consistently desensitize semantic pathways resulting in a reduction of following subliminal semantic priming.

### Materials and methods

#### Subjects

Participants were 25 healthy, right-handed (according to handedness test by Oldfield, 1971), native German speakers. The data of three participants had to be excluded due to technical problems in data acquisition. The remaining 22 subjects (nine men and 13 women) were in the age range of 19–26 years, with a mean of 21.95 years. All participants had normal or corrected-to-normal vision and none reported any history of neurological or psychiatric disorders. None of these participants took part in the pilot studies or in Experiment 1.

#### Material and procedure

***Induction task.*** The semantic induction task was the same task as in the first experiment (living/non-living decision). For selecting the stimuli for the phonological letter induction task, a stimulus set of 200 words was tested in a pilot study with an independent sample of eight subjects (five women, three men; on average 23.5 years old). The stimuli for the phonological letter induction task consisted of German words, which were written in lowercase letters with an uppercase letter at different positions. The position of the uppercase letter within each word was evenly distributed across all stimuli. First and last position was always written in lower case to avoid oversimplification of the task. Half of the word included a vowel as capital letter (e.g., hErbst, Engl. autumn; wurzEl, Engl. root). The other half contained a consonant as capital letter (e.g., kaFfee, Engl. coffee; woLke, Engl. cloud). The participants' task was to decide whether the uppercase letter within the presented word was a vowel or a consonant.

Based on this pilot study, a final set of 80 words was selected for the phonological letter induction task in such a way that both induction tasks (semantic and phonological letter) were matched for response times as closely as possible. Thus, RTs and ERs did not significantly differ between the phonological letter induction task and the semantic induction task (661.51 vs. 696.12 ms, *p* > 0.09; 2.3 vs. 2.5%, *p* > 0.64). Besides the different phonological induction task (phonological letter decision), experimental design was identical to Experiment 1. Again, different word sets were used for the induction tasks and the lexical decision task.

### Results and discussion

#### Masked word identification task

As in Experiment 1, we assessed the visibility of the masked primes in an identification test following the priming phase. Identification performance was distributed around the chance level of 50% (mean phonological letter = 48.0%, mean semantic = 49.5%), which is expected by mere guessing. A repeated-measures ANOVA on d′ measures (for details see Experiment 1) with the within-subject factors induction task and semantic relatedness revealed no significant differences between conditions (all *F*s < 3.93, all *p*s > 0.061). Thus, it can be excluded that backward priming rendered the masked prime words partially recognizable. Additionally, masked primes were not differentially visible following the phonological letter (*d*′ = −0.04) and semantic (*d*′ = 0.15) induction task. All d′ did not significantly deviate from zero (*p* > 0.22). Additionally, a correlation between d′ and semantic priming was close to zero (*r* = 0.13) and not significant (*p* > 0.56). As in Experiment 1, we applied a median split to divide the participants according to their prime visibility index d′ into a “low visibility” and a “high visibility” visibility group in the semantic induction task condition (Ortells et al., 2012b). In the semantic induction task condition, the high visibility group showed a d′ mean of 0.48, which was reliably above chance, *t*_(10)_ = 4.06, *p* < 0.01, whereas the low visibility group had a d′ mean of −0.17, which did not reliably differ from zero, *t*_(10)_ = −1.85, *p* > 0.09. Additionally, a *t*-test for independent samples on semantic priming performed for group (high vs. low visibility) revealed no significant difference in the magnitude of semantic priming, *t*_(20)_ = 1.23, *p* > 0.23, two-tailed.

#### Induction tasks

Analysis of the data of the induction task was identical to Experiment 1. Six hundred eighteen trials from the whole data set were rejected as outliers (4.4%). An ANOVA with repeated-measures was calculated on mean RT and ER that included the factor induction task. RT in the phonological letter induction task was significantly shorter compared with RT in the semantic induction task (660.81 vs. 752.15 ms), [*F*_(1, 21)_ = 17.76, *p* < 0.0001, η^2^ = 0.46]. ER analysis did not yield any significant effects.

#### Masked semantic priming

**Figure 3:**
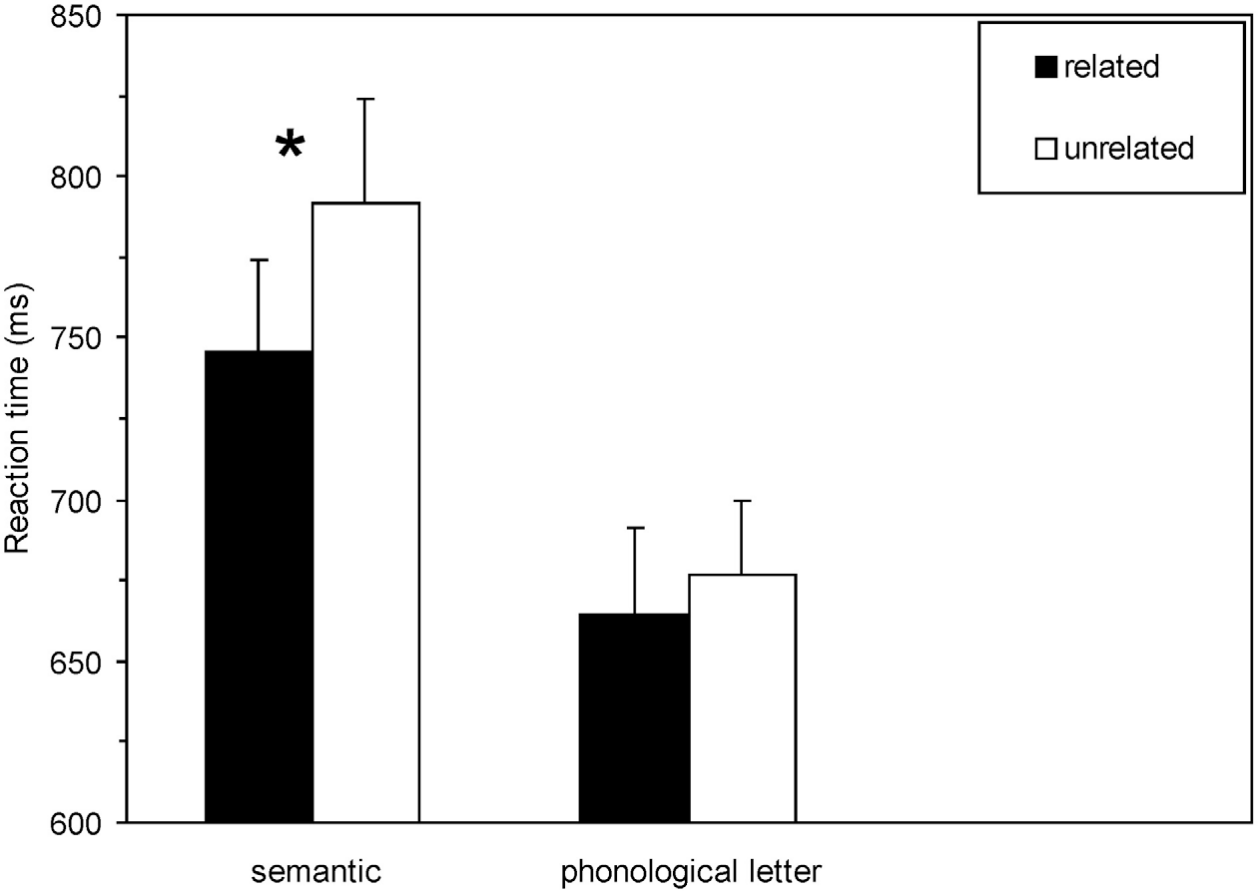
**Experiment 2.** Mean lexical decision latencies (*n* = 22) as a function of semantic relatedness and induction task. Related = semantically related prime target pairs; unrelated = semantically unrelated prime target pairs. Please note the significant interaction (*p* < 0.01) between induction task and semantic relatedness due to attenuated priming subsequent to the phonological letter induction task compared to the semantic induction task. The asterisk indicates significant priming effects (*p* < 0.05).

Analysis of the data of the masked priming task was identical to Experiment 1. Two hundred ninety-six trials from the whole data set were rejected as outliers (2.1%). A repeated-measures ANOVA with the within-subject factors induction task and semantic relatedness was performed on mean RT of correct responses as dependent variable. The main effect of induction task as well as the main effect of semantic relatedness were significant, [*F*_(1, 21)_ = 12.8, *p* = 0.002, η^2^ = 0.38]; [*F*_(1, 21)_ = 32.58, *p* < 0.0001, η^2^ = 0.61], respectively. Most importantly, the critical interaction involving induction task and semantic relatedness was also statistically reliable, [*F*_(1, 21)_ = 8.12, *p* = 0.01, η^2^ = 0.28]: masked semantic priming was larger following the semantic induction task compared to the phonological letter induction task, 45.9 vs. 12.8 ms, respectively (see Figure 3).

Fisher LSD tests demonstrated significant semantic priming (faster RT to semantically related than to unrelated targets) only subsequent to the semantic induction task, *p* < 0.0001, but not subsequent to the phonological letter induction task, *p* > 0.14. An ANOVA on ER with the within-subject factors induction task and semantic relatedness revealed a main effect for induction task, [*F*_(1, 21)_ = 12.04, *p* = 0.002, η^2^ = 0.36], and a main effect for semantic relatedness, [*F*_(1, 21)_ = 35.6, *p* < 0.001, η^2^ = 0.63]. Following the semantic induction task, participants committed more errors in the lexical decision task than following the phonological task (11.31 vs. 7.61%). More errors were committed in the non-related condition compared to the related condition (11.93 vs. 6.99%).

#### Conjoint analysis of Experiments 1 and 2

In order to compare the modulation of subliminal semantic priming by induction tasks between experiments, we conducted a conjoint analysis on the semantic priming effects (RT unrelated minus related). A repeated-measures ANOVA with the within-subject factor induction task and the between-subject factor experiment (Experiments 1 and 2) yielded a main effect of induction task indicating significantly more subliminal semantic priming in the semantic induction task condition (37.03 ms) compared to the phonological induction task condition (11.5 ms), [*F*_(1, 42)_ = 6.61, *p* = 0.01]. However, neither the factor experiment nor the interaction between group and induction task did reach significance, [*F*_(1, 42)_ = 1.62, *p* = 0.21], [*F*_(1, 42)_ = 0.58, *p* = 0.45], respectively. Hence, modulation of unconscious semantic priming by the induction tasks did not significantly differ between experiments.

The second experiment served to investigate whether the phonological letter induction task, which strongly focuses attention to the phonology of single letters and discourages word reading, more clearly abolishes following masked semantic priming compared to the phonological word induction task of Experiment 1. We found a differential modulation of masked semantic priming by the two induction tasks as shown by the significant interaction between induction task and semantic relatedness: subliminal semantic priming was found to be significant subsequent to the semantic induction task (45.9 ms), but was attenuated following the phonological letter induction task (12.8 ms). Thus, attention to the phonology of single letters within a word strongly desensitizes semantic processing pathways and consequently decreases subsequent subliminal semantic priming more pronounced compared to Experiment 1. It should be noted however that the conjoint analysis of both experiments did not reveal a significantly different priming pattern suggesting that the modulation of subliminal semantic priming by the semantic vs. phonological induction tasks was comparable in both experiments. Together with the findings of Experiment 1, Experiment 2 further specifies the attentional task set conditions for unconscious semantic priming to occur: the results suggest that unconscious semantic processing depends on an activated semantic task set while a task set focused on phonology attenuates unconscious semantic priming.

## General discussion

In the present study, we further investigated the attentional boundary conditions of unconscious semantic processing with our previously developed induction task paradigm (Kiefer and Martens, 2010; Martens et al., 2011). We addressed the question whether phonological induction tasks reduce subliminal semantic priming compared to semantic induction task similarly to the effects observed previously with perceptual letter classification tasks (Kiefer and Martens, 2010). This would demonstrate that an attentional orientation toward semantics is important for unconscious semantic processing to occur. Furthermore, we tested whether the effects of the phonological induction tasks on subsequent priming are comparable whether the attentional focus is set on phonological processing of the entire word vs. single letters of a word. Across two experiments, we varied the nature of the phonological induction task and contrasted the influence of a phonological word decision with that of a phonological letter decision task on subsequent masked semantic priming effects.

In line with our attentional sensitization model (Kiefer and Martens, 2010), the present study shows a differential modulation of masked semantic priming by semantic and phonological induction tasks: in the first experiment, we found subliminal semantic priming effects only following the semantic induction task, but not following the phonological word induction task, although this difference in priming between induction tasks was not statistically significant. This subliminal priming pattern as a function of induction task was replicated and even more pronounced in the second experiment with the phonological letter induction task, where the interaction between semantic relatedness and induction task was statistically significant. Again, unconscious semantic priming effects were only observed subsequent to the semantic induction task, but not subsequent to the phonological letter induction task.

As in both experiments reliable subliminal semantic priming was only obtained following the semantic, but not following one of the phonological induction tasks, this indicates that an attentional orientation toward semantics is necessary for subliminal semantic priming to occur. The present results therefore nicely agree with findings from our previous study (Kiefer and Martens, 2010), in which a perceptual letter induction task, which required participants to pay attention to shape of the letters of a word, produced similar results. Hence, attention to both perceptual and phonological features reduces following subliminal semantic priming. According to the attentional sensitization model of unconscious cognition (Kiefer, 2007; Kiefer and Martens, 2010), a semantic induction task results in a relative sensitization of semantic pathways whereas non-semantic phonological or perceptual tasks result in a relative desensitization of semantic pathways. As a consequence of this differential attentional configuration, the unconsciously perceived masked prime words are only semantically processed and can elicit priming effects when a semantic task set is active.

It should be noted that masked semantic priming was attenuated subsequent to both the phonological word and letter induction tasks, although they differed with regard to the overall difficulty level: RT to the word phonological induction task in Experiment 1 was slower compared with the semantic induction tasks whereas RT in response to the phonological letter induction task in Experiment 2 was faster than to the semantic induction task. This RT difference reflects the fact that the phonological word induction task of Experiment 1 required reading the entire word, which is more difficult and time consuming than processing of single letters/phonemes in the phonological letter induction task of Experiment 2. Hence, most importantly, the reduction of masked semantic priming was not only observed subsequent to the difficult word induction task (Experiment 1), but also, even more compelling, subsequent to the relatively easy phonological letter induction task (Experiment 2), which presumably imposes less demands on attentional capacity than the semantic induction task (for the influence of attentional capacity on masked semantic priming, see Martens and Kiefer, 2009). Hence, in line with similar findings in our earlier study with perceptual inductions tasks (Kiefer and Martens, 2010) our results show that non-semantic task sets attenuate semantic priming irrespective of the difficulty level of the tasks that are used to induce them.

Although the results of the two experiments are clearly in line with the notion of attentional sensitization of unconscious processing, two alternative explanations of the induction task effects on subliminal priming are conceivable. It could be argued that the observed modulation of masked priming by previously performed induction tasks reflects interference effects of the activated words presented in the induction task on the subsequent masked prime. This would result in a weaker semantic prime activation. However, when arranging the words within one trial (word in the induction task, masked prime, and target word), we controlled that words in the induction task and the masked lexical decision task (prime and target word) were not semantically related. Semantic competition typically occurs only though for close associates, but not for unrelated concepts (Humphreys et al., 1988; Carr and Dagenbach, 1990). Thus, the attenuation of semantic priming subsequent to the phonological word/letter induction task cannot be attributed to such bottom–up semantic interference effects. As a further explanation of this result pattern, it could be purported that response congruency effects between the induction task and the masked lexical decision task are responsible for the observed modulation of masked priming by previously performed induction tasks. However, response requirements (response finger) and experimental condition were entirely counterbalanced, thereby excluding the possibility that any effects of response congruency could have compromised our findings. Furthermore, one could argue that our vowel/consonant decision task does not involve phonological processing, but depend on visual orthographic processing similar to our previous studies (Kiefer and Martens, 2010; Martens et al., 2011). This possibility is, however, unlikely because vowel/consonant classification of phonemes of a word is typically considered as phonological task (van Turennout et al., 1997, 1999; Abdel Rahman and Sommer, 2003; Abdel Rahman et al., 2003). Furthermore, the vowel/consonant distinction is primarily based on phonology and only secondary indexed by orthography (Levelt, 1989). Nevertheless, it would be interesting to replicate our findings in future studies with other non-semantic induction tasks that involve more than one letter and focus on phonology or syntax (e.g., rhymes and gender).

There were subtle differences in the effects of the phonological word (Experiment 1) and letter induction task (Experiment 2) on subsequent masked priming, although, they were not statistically significant in the conjoint analysis of both experiments: the reliable reduction of subliminal semantic priming by the phonological letter induction task compared with the semantic induction task indicates that attention to single letters/phonemes of a word strongly disrupts subsequent semantic processing of unconsciously presented primes. The phonological word induction task that permits word reading also reduced subsequent subliminal semantic priming, but statistically less reliable compared with the phonological letter induction task. As outlined above, dual route models of reading (Riddoch et al., 1988; Seidenberg and McClelland, 1989) suggest that word reading includes both semantic and non-semantic pathways. The two alternative processing pathways underlying word reading may lead to considerable interindividual variability with regard to the specific nature of the phonological task set activated by the phonological word induction task (non-semantic vs. semantic route). This may result in a less reliable reduction of subliminal semantic priming (priming difference between induction tasks: 18 ms) compared with the phonological letter task, which unequivocally activates a non-semantic task set and thus consistently desensitizes semantic pathways (priming difference between induction tasks: 33 ms). It should also be noted that the overall magnitude of priming was numerically, albeit not significantly, smaller in Experiment 1 than in Experiment 2, particularly following the semantic induction task, possibly due to interindividual differences in priming (Kiefer et al., 2005). The smaller magnitude of priming may have additionally decreased the statistical power for detecting priming differences between induction tasks in Experiment 1.

The present attentional effects on unconscious semantic priming are compatible with earlier studies on the effects of prime tasks on visible semantic priming. These studies showed that letter search on the prime attenuated semantic (Smith et al., 1983; Besner et al., 1990; Chiappe et al., 1996) or phonological priming (Ferguson and Besner, 2006; Kahan et al., 2006). Our study considerably extends this earlier work: in our experiments semantic processing was investigated under subliminal and thus exclusively automatic processing conditions. In these earlier studies on prime task effects, in contrast, primes were presented visibly and priming effects presumably also reflect controlled semantic processes (Posner and Snyder, 1975; Neely, 1991). The present results show that even under purely automatic processing conditions, semantic priming is susceptible to attentional top–down control as predicted by our attentional sensitization model. Hence, automatic semantic processing and the notion of attentional control is not a contradiction as previously thought (for a similar argument see, Moors and De Houwer, 2006).

The subliminal priming pattern in our study therefore helps to resolve the fierce debate about the automaticity of semantic processing. As outlined in the introduction, it has been suggested that semantic processing requires controlled access to conceptual meaning because semantic priming is sensitive to attentional manipulations (McCarthy and Nobre, 1993; Henik et al., 1994; Chiappe et al., 1996; Kellenbach and Michie, 1996; Duscherer and Holender, 2002). In contrast, there is also numerous evidence that semantic processing can occur unconsciously in an automatic fashion (Deacon et al., 2000; Kiefer and Spitzer, 2000; Rolke et al., 2001; Kiefer, 2002; Heil et al., 2004; Grossi, 2006; Kiefer and Brendel, 2006; Ortells et al., 2012b). In line with our attentional sensitization model, the present work on top–down effects on masked semantic priming reconciles these seemingly discrepant previous findings by demonstrating how specific attentional task sets enhance or attenuate subliminal semantic priming. We show that automatic semantic processing depends on an attentional configuration of the cognitive system that must entail access to word meaning. The induction task paradigm, combining a task for inducing task sets with a subsequent masked priming paradigm, used in this research has been proven as a powerful tool for studying attentional control of automatic processes at a fine-grained level.

In conclusion, the present subliminal priming study has identified important attentional boundary conditions for unconscious semantic processing to occur. We showed that unconscious semantic processes elicited by masked primes depend on a previously activated semantic task set that sensitizes semantic pathways. In contrast, phonological task sets whether they focus on word phonology or on the phonology of single letters attenuate semantic priming. This suggests that not only perceptual task sets that focus on the shape of single letters as shown previously, but also other non-semantic tasks sets (here: phonological word or letter task sets) desensitize semantic pathways and reduce unconscious semantic priming. Our results therefore demonstrate the generality of attentional control of unconscious semantic priming and help to accommodate seemingly incompatible findings regarding the automaticity of semantic processing.

### Conflict of interest statement

The authors declare that the research was conducted in the absence of any commercial or financial relationships that could be construed as a potential conflict of interest.
